# Monobrominated Camphor in Summer Complaints of Children

**Published:** 1877-08-20

**Authors:** 


Monobrominated Camphor in Summer Complaints of Children.—Dr. H. W. Taylor, in *Med. Rev.*, speaks in exalted terms of monobrominated camphor in cholera infantum and diarrhœas of children. His reports show an unprecedented success with this remedy. He uses it in what he calls the first decimal trituration (homeopathic)—one grain triturated with ten grains of the sugar of milk. Of this he gives one grain in oft repeated doses. He remarks, "It is the best showing ever made by any drug, or combination of drugs, in this terrible disease."

